# Gout and comorbidity: a nominal group study of people with gout

**DOI:** 10.1186/s13075-017-1416-8

**Published:** 2017-09-15

**Authors:** Jasvinder A. Singh

**Affiliations:** 1 Medicine Service, Birmingham VA Medical Center, Birmingham, AL USA; 2 Department of Medicine at School of Medicine, and Division of Epidemiology at School of Public Health, University of Alabama at Birmingham, Faculty Office Tower 805B, 510 20th Street S, Birmingham, AL 35294 USA

**Keywords:** Gout, Comorbidity, Qualitative, Nominal group, NGT

## Abstract

**Background:**

Comorbidities are common in patients with gout, yet qualitative research is lacking. The study objective was to examine the impact of gout on comorbidities.

**Methods:**

Nine nominal groups were conducted. Patients with gout discussed and rank-ordered their concerns in response to the question, “How does gout or its treatment affect your other conditions and their treatment?”

**Results:**

Nine nominal groups had 45 gout patients, with mean age 61 years (standard deviation (SD) 10.7) and mean gout duration 14.9 years (SD 12). Of these, 62% were men, 45% African-American, 51% married and 63% were currently using allopurinol. The most frequently cited highly ranked concerns among the nine nominal groups were: (1) interaction of gout medication with medications for other medical conditions (three groups); (2) worsening of other medical comorbidities, including hospitalizations (seven groups); (3) worsening of anxiety and depression (three groups); (4) significant dietary changes for gout that contrasted with diet for other conditions (three groups); (5) new diseases diagnosed due to gout (three groups); (6) irreversible joint damage (three groups); (7) inability to exercise and weight gain (four groups); and (8) gout misdiagnosed as another health condition (three groups). Other domains ranked highly were: (1) impact of gout on daily life and activities, including the ability to work and social activities (six groups); (2) medication side effects, real and perceived (nine groups); (3) weight loss due to gout related to frequent flares (one group); and (4) cost and burden (three groups).

**Conclusions:**

Gout and the medications used for its treatment have a significant effect on comorbidities and their management. These findings provide insights into potential targets for improving outcomes in patients with gout.

## Background

Gout affects 8.3 million Americans and is the most common inflammatory arthritis in adults [1]. Gout is associated with metabolic syndrome and obesity [2]. Not surprisingly, medical comorbidities such as heart disease, hypertension, diabetes mellitus, heart failure, and sleep apnea are more common in patients with gout compared to those without gout [3–8]. Lower quality of life (QoL) has been reported in patients with gout [6, 9–11]. In a previous qualitative study of nominal groups assessing QoL in patients with gout, comorbid conditions were identified as important contributors to QoL in gout [12]. Comorbidities were also associated with more frequent gout flares in patients with gout [5]. Thus, comorbidities are common in patients with gout and comorbidities are known to have a negative association with QoL.

On the other hand, little is known about how gout affects the comorbidities and their outcomes. Most qualitative research in gout has focused on physician or patient knowledge, attitudes, and treatment adherence [13–17], or the impact of gout on QoL [15, 18, 19]. Published qualitative research has focused on Caucasian men, while gout is more common and more severe in African-Americans than in Caucasians (5% vs. 4% in the USA) [1], and who also have more functional limitations [20] and worse QoL [20]. Similarly, most gout studies include predominantly men, and women are under-represented. Therefore, qualitative studies are needed with adequate representation of African-Americans and women with gout that focus on the effect of gout on comorbidity management and outcomes.

Corbin and Strauss developed the trajectory model further refined by Charmaz and others that includes three components – body (organ system and function), biographical time (explicit narrative that gives meaning and purpose to a person’s life) and conceptions of self (role identity, social identity, etc.), i.e., the BBC chain [21, 22]. A person enjoys a sense of health and wellbeing only when body, biographical time, and conceptions of self are in balance, interactively stabilizing and reinforcing one another [21–23]. By its effect on comorbidities, I hypothesized that gout might destabilize this BBC chain due to associated progressive loss of self from body failures (worsening of kidney or heart disease), failed performance and social isolation (suboptimal management of multiple comorbidities leading to an inability to function normally) [24]. Therefore, the aim of this formative study was to assess the effect of gout (and its treatment) on comorbidity, considering all aspects, using the nominal group technique (NGT), and assessing if this fits our proposed theoretical model, the trajectory model.

## Methods

### Study sample, NGT sessions, and analyses

Consecutive patients with at least one visit to the community-based clinic at the University of Alabama at Birmingham (UAB) between January 2016 and February 2017 for gout, identified by the presence of an *International Classification of Diseases, ninth revision, common modification* (ICD-9-CM) code, 274.xx, were invited for study participation. African-Americans and women were oversampled. Free parking, refreshments during the session, and a US$30 check were provided to each participant. The UAB Institutional Review Board (IRB) approved the study.

The NGT is a variant of the traditional focus group that taps the experiences, skills, and views of the participants. NGT has been used successfully in understanding participant views in several medical conditions and allows an in-depth examination of a single question [25–31]. NGT promotes even participation. NGT methods help in developing an inclusive list of issues related to a specific question followed by soliciting participant feedback on the relative importance of this list using a rank-ordering procedure [28, 32]. This exercise facilitates representation of the implicit views of the group.

Patient nominal group sessions were conducted to understand the impact of gout on comorbidity. Each nominal group session was led by an experienced NGT moderator (JAS) [12, 33] and lasted 1–1.5 hours. After informed consent, everyone gave brief introductions. Each participant was provided with the study question printed on top of a blank sheet of paper, which was also written on a flipchart: “How does gout or its treatment affect your other conditions and their treatment?” Participants were asked if the main question was clear and if they had any questions about it, which were addressed by the NGT moderator before starting each session. The NGT participants independently generated as many words or short phrases as possible in response to the question on a sheet of paper. All responses identified by each participant were recorded verbatim on a flip chart in large letters by the NGT moderator (JAS), so that they were visible to participants. Participants discussed and elaborated each response and combined responses that were similar, where appropriate. All participants rank-ordered the three responses deemed most important with scores from 1 to 3 on index cards, 3 being the highest rank score. A rank order was created for each nominal group based on total scores, with the highest score corresponding to the top rank.

All NGT discussions were recorded and fully transcribed by an administrative assistant (DF) and transcriptions were examined to identify discussion related to each response, which led to the creation of a comprehensive list of statements. Responses were compared to determine overlap and saturation of themes was confirmed.

## Results

### Study participant characteristics

Nine nominal groups were conducted with 45 gout patients (range between 2 and 10 people per nominal group) with a mean age of 61 years (standard deviation 10.7), of which 62% were men, 45% African-American, 51% were married and 45% had a college degree (Table 1). Most patients also had at least one additional comorbidity (specific data not collected). Only 9% were on colchicine only and 2% were not receiving any gout medication; the remaining patients were using allopurinol, febuxostat or both. Almost two thirds were currently using natural supplements and 39% had had two or more gout flares in the last 6 months. Of the nine nominal groups, three consisted of all men, and one consisted of five women and one man (female predominant). There were no significant differences in mean age (standard deviation) and gout duration by race/ethnicity or gender, respectively: whites vs. African-Americans, 62.9 (11.0) vs. 61.8 (5.2) (*p* = 0.68) and 17.9 (11.6) vs. 12.3 years (9.3) (*p* = 0.09); men vs. women, 60.1 (11.8) vs. 62.3 (8.4) (*p* = 0.50) and 17.0 (11.1) vs. 11.2 years (9.5) (*p* = 0.08). A saturation of themes was achieved.

**Table 1:** Demographics of nominal group participants (n = 45)

	Number (percentage)^a^
Age in years, mean (SD)	60.9 (10.7)
Sex, male (%)	28 (62%)
Disease duration in years, mean (SD)	14.9 (12)
Race/ethnicity	
White	22 (49%)
African-American	20 (45%)
Hispanic	1 (2%)
Asian	2 (4%)
Education level	
Less than high school	1 (2%)
High school graduate	7 (15%)
Some college or technical/vocational training	16 (35%)
College Bachelor degree and beyond	21 (46%)
Marital status	
Divorced	7 (15%)
Domestic partnership	1 (2%)
Married	23 (51%)
Separated	4 (9%)
Single	7 (15%)
Widowed	3 (7%)
Employment status^b^	
Employed	5 (11%)
Homemaker	1 (2%)
Retired	21 (46%)
Self-employed	5 (11%)
Unable to work	12 (26%)
Current medications to treat gout^c^	
Allopurinol	8 (17%)
Allopurinol, pain medication (NSAID or analgesic or narcotic)	3 (7%)
Allopurinol, colchicine (with/without pain medication)	13 (28%)
Allopurinol, colchicine, prednisone (with/without pain medication)	5 (11%)
Colchicine alone	4 (9%)
Allopurinol, febuxostat, colchicine, prednisone, pain medication	2 (4%)
Febuxostat (with/without colchicine and/or prednisone)	7 (15%)
None	1 (2%)
Current use of natural supplements^d^	
None	15 (33%)
Cherries	1 (2%)
Cherry extract or concentrate	5 (11%)
Cherry juice	6 (13%)
Multivitamin	8 (17%)
Vitamin C	3 (7%)
Others	2 (4%)
Number of gout flares in the last 6 months^e^	
0	12 (36%)
1	7 (21%)
2	1 (3%)
3–5	5 (15%)
≥ 6	7 (21%)

^a^Number (percentage) unless otherwise specified

^b^One person did not respond to this question

^c^Two people did not respond to this question

^d^Six people did not respond to this question

^e^One person did not respond to this question; since the question was added from the third nominal group onwards, the number of total responses was 33

The top themes/concerns that emerged from each nominal group are listed in Table 2 (also see Appendix 1 for details). These themes mapped to body failure, the limitation of identity-relevant performances with a progressive loss of self and/or to social isolation. Despite specification of the question relating to the effect of gout or its treatment on their comorbid conditions and their treatments, several patients and groups chose to rank the overall effect of gout or its treatment on daily life, since they considered the overall wellbeing just as important as (or more important than) a specific disease, i.e., heart disease or hypertension. For the sake of clarity, the themes from nominal groups were divided into (1) the effect of gout/treatments on comorbidity and their treatment and (2) the general effect on overall wellbeing (in the subsequent sections). Letters (A to L) at the beginning of each theme correspond to those identified in the consecutive nominal groups (see Table 2), and therefore are not in a sequential order in each section.

**Table 2:** Main themes ranked by nominal group technique (NGT) participants

Theme	Score
NGT1, 3 people: 3 male; 3 African-American; 18 votes	
A. When gout attacks it affects my movement, to walk and work	9
B. Before I got on the treatment, that I am on right now, the medicine made me sick (allopurinol associated nausea and allergic reaction)	5
C. Negative response to blood pressure treatment	2
D. Someone I know lost a lot of weight due to active gout that affected his other conditions	1
E. During a gout attack, gout test and potassium levels went high and had to be treated	1
NGT2, 10 people; 4 male, 6 female; 4 white, 5 African-American, 1 Asian; 60 votes	
A. It affects everyday body and brain functions, movement, physical or mental; it makes me less independent	18
K. Problem with misdiagnosis: It took a longer time frame for diagnosis of the actual problem in my joints, which is psoriatic arthritis, not gout	7
F. More Depression/anxiety due to gout	4
E. Pain of gout increases your blood pressure and messes with the heart condition	4
G. Specific diet advise differs between conditions, leading to contradictions, low protein for gout vs. high protein for other conditions; With restriction for diabetes and gout, what is left to eat?	4
B. I have kidney problems they had to take me off colchicine, since it was causing kidney problems; I was put on colchicine, caused gastric problems, has to be stopped	10
G. I am diabetic, trying to avoid carbs; proteins that want to take to help regulate my diet, are problematic with gout; I was prescribed fish oil for lowering cholesterol but can’t take that due to gout	3
J. Had problems with weight gain due to fluid retention due to gout; excessive weight gain due to steroids caused sleep apnea	3
E. I have pulmonary hypertension, heart failure, it does not help	3
C. It interferes with other medications and makes me tired- I take so much medicine	2
H. Kidney stones were calcium oxalate only, now they are urate and calcium oxalate since I got gout	2
NGT3, 5 people; 2 male, 3 female; 4 white, 1 African-American; 30 votes	
I. My joints are so sore now all the time, wondering if they are damaged due to (gout) flares	8
B. Some of the gout medications such as prednisone can cause weight gain, diabetes and affect eyesight; NSAIDs cause bleeding	8
E. I did not have high blood pressure until I was diagnosed with gout- not sure it caused it	9
J. Having gout makes me immobile, which makes it hard for me to loose weight	4
K. New diagnosis of rotator cuff for the last 3 weeks: Gout caused it to be misdiagnosed	1
NGT4, 5 people; 4 male, 1 female; 1 white, 3 African-American, 1 Asian; 30 votes	
B. Gout treatment, in particular, prednisone caused weight gain; acid reflux due to pain medications for gout	12
H. Gout putting you at risk for other conditions	7
J. Immobility and inability to exercise due to gout	5
K. Gout being confused with other conditions – I was tested for leg clots many times	4
E. Higher dose of allopurinol made my diabetes worse	2
NGT5, 4 people; 3 male, 1 female; 3 white, 1 African-American; 24 votes	
B. Gout medicines over long period time cause renal failure, stomach upset, stomach ulcers, hip necrosis	19
H. Gout crystals caused me the “kidney problem”- kidney damage – “They are like razor knives, they cut your tissue – your kidney, other organs”	3
L. The medication I take caused me to get my liver checked often	2
NGT6, 7 people; 7 male; 4 white, 2 African-American, 1 Hispanic; 42 votes	
A. It affects my ability to work- “No body goes to work with gout, you have to be wheel-chaired in”	12
L. Very expensive disease to control- Colchicine went from a few dollars a month to high price and co-pay overnight	8
B. Side effects- Indocin can mess up your liver; Colchicine and me don’t agree	9
J. It affects my ability to do my work-out regimen, I work out 3-4 times a week	5
F. It affects my ability to concentrate and stay focused- When I am in such abstract pain, I can’t do anything	3
L. Frequent regular visits to the doctor and the lab to monitor various levels of everything to adjust medications - Access to doctors is an issue, when I have to travel, if something happens, whom do I call?	3
E. It totally incapacitates me and causes me to be hospitalized	1
I. Used to have Achilles tendinitis which was due to gout- Had one surgery for my broken ankle and then others due to arthritis I had due to my gout crystals damaging my joints	1
NGT7, 6 people; 1 male 5 female; 3 white, 3 African-American; 36 votes	
A. Since you can not walk because of your pain, you are going to gain weight, and it will impact heart, lungs and body conditioning	19
F. Makes me depressed	7
E. It affects my eczema, when it is (gout flare) in my foot, the foot is itching all the time, and it bleeds if I itch it; it affects my blood pressure	7
B. A lot of gout medications affect kidneys and liver, especially the pain medication, so you can’t take a lot of it	3
NGT8 2 people; 1 male, 1 female; 2 African-American; 12 votes	
B. Gout medication – I was allergic to allopurinol; ibuprofen and Aleve scarred my kidney	7
C. Some of the medicines I can not take with my gout medicine- they interact- Gleevac and colchicine	5
NGT9; 3 people; 3 male; 3 white: 12 votes (1 patient left before the vote)	
H. Gout is causing other health conditions- I think it (can) lead to heart disease, diabetes, cholesterol problems, artery stiffness and clogging	4
E. It has affected sleep and that affects my overall health- Flares as well as when the gout hurts all the time	2
B. At the back of my mind, I am concerned about the fact that the medicine is affecting my body	1
A. Limits going to work, activity, socializing and going out	5
I. Joints wear out quicker because of gout- I had two joints repaired, in wrist and ankle	0

Each patient gave 3 points to the highest rank, 2 points to the second highest and 1 point to the third highest rank. In most cases, the number of total votes is the number of patients × 6, except in one case when the patient left before voting, due to transportation issues (NGT9)

### The impact of gout or gout treatment on comorbidities and their management

#### Interaction of gout medications with medications for other medical conditions (C)

Three nominal groups ranked this among their top concerns. Participants reported interaction of gout medications with: (1) medications for their hypertension, making them less effective; (2) Gleevec (the market name for imatinib, an anti-cancer medication), with an inability to take Gleevec and colchicine together; (3) medications for concomitant diseases, leading to more fatigue; (4) grapefruit juice, since they were instructed not to take gout medications with grapefruit juice.

#### Worsening of other medical comorbidities, including hospitalizations (E)

Seven patient groups listed this among their top concerns. Patients described that gout flare and associated pain increased their blood pressure and heart rate and worsened their heart condition, eczema, and other medical conditions. Pain medications such as ibuprofen worsened heart conditions; and allopurinol interfered with diabetes mellitus control. Participants were hospitalized with gout flares that also affected their other conditions.

#### Worsening of anxiety and depression (F)

Three patient groups listed this among their top concerns. Participants experienced worsening of depression and anxiety due to gout. Acute gout flare interfered with the ability to stay focused and increased the risk of depression.

#### Significant changes in diet with dietary restrictions that contrast with diet for other chronic conditions (G)

Three patient nominal groups listed this among their top concerns. Patients wanted to go on high protein diet for weight loss to help diabetes mellitus and heart conditions, or eat more fish or take fish oil for heart disease prevention, but these diets seemed to worsen gout. Many believed that the cabbage or dry beans that they wanted to eat for a heart-healthy diet increased the risk of gout flare, therefore the presence of gout interfered with the intake of the “healthy” foods that they liked for better comorbidity management and/or prevention of associated compliations.

#### New disease diagnosis due to gout (H)

Three patient nominal groups listed this among their top concerns. Patients had new diagnoses of kidney disease, hypertension, heart disease, sleep apnea, and kidney stone disease (urate stones and calcium stones), which they did not have before the diagnosis of gout. They believed that gout was the reason for them to have these new diseases. Patients likened the urate crystals to “razor knives that cut through the tissues”.

#### Irreversible joint damage due to arthritis and joint/tendon problems (I)

Two patient nominal groups listed this among their top concerns. Patients experienced progressive joint symptoms and joint destruction, which put people at risk of joint replacement surgery, and led to progressive symptoms over time, sometimes leading to long hospitalizations.

#### Inability to exercise and weight gain (J)

Four patient nominal groups listed this among their top concerns. The gout flare pain immobilized people, and made it impossible for them to exercise and work out, which they needed to do regularly for a better management of other chronic conditions.

#### Gout confused with and diagnosed as another health condition (K)

Three patient nominal groups listed this among their top concerns. Patients reported that their gout had been misdiagnosed as muscle sprain, rotator cuff tendinitis, or a leg clot.

Domains C, E, F, G, H, and K mapped to body failure. Domains I and J mapped to body failure and failed performances.

### The impact of gout or its treatment on daily life

#### Impact of gout on daily life and activities, including the ability to work and social activities (A)

Six of the nine groups listed this among their top concerns. Participants reported that gout interfered with mobility and independence and affected their ability to function, go shopping, doing yard work, housekeeping, driving, going out, or doing physical therapy for back pain or after joint replacement. Gout also influenced the ability to work, making patients immobile during a flare and making them need a cane and other assistive devices: “Nobody goes to work with gout”. Gout made it difficult for participants to play with grandchildren, limited social life, made them quit hobbies, and forced some to not take long vacations or travel far.

#### Medication side effects, real and perceived (B)

All nine groups listed this among their top concerns. Most groups reported real side effects of treatments they experienced, and in many cases, the medication had to be discontinued due to the occurrence of the side effect. Side effects associated with medication reported by participants were: (1) allopurinol: nausea, allergic reaction, itching, rash, headache, or brain fog; (2) colchicine: problems with calcium levels, kidney function, diverticulitis, brain fog, dry mouth, stomach upset, or sun sensitivity; (3) non-steroidal anti-inflammatory drugs (NSAIDs): diverticulitis, leg swelling, acid reflux, kidney disease, gastrointestinal (GI) bleed, liver problems, or diarrhea; and (4) corticosteroids: weight gain, GI upset, leg swelling, or hip necrosis. One group also ranked high the worry about long-term side effects of various gout medications even in the absence of any experienced side effects.

#### Weight loss due to gout, not related to flares (D)

One patient group listed this among their top concerns. Active gout with frequent flares was associated with weight loss.

#### Cost and burden (L)

Three patient nominal groups listed this among their top concerns. Patients complained that some medications for gout, such as Colcyrs (the market name for colchicine) were very expensive. They also found frequent physician and laboratory visits for monitoring burdensome.

Domains B, D, and L mapped to body failure. Domain A mapped to body failure, failed performances and social isolation.

### Gender differences in the impact of gout on the lives of patients

No observable differences in ranking of themes by gender were noted, since similar themes were ranked highly by male vs. female-predominant nominal groups.

## Discussion

This qualitative study focused on the impact of gout and its treatment on other health conditions (comorbidities) and their management. We achieved an adequate representation of African-Americans and women with gout, two understudied populations in gout. Several findings merit further discussion and add to the current knowledge, in the absence of previous qualitative work on this topic. We conducted nominal groups until saturation was achieved. We believe that the small sample size for three nominal groups (n = 2–3 people in each group), the global and broad nature of our question and the diverse socio-demographic composition of our sample with oversampling of African-Americans and women, may have led to the current need for more nominal groups (nine) than the usual number (4–5 nominal groups), before saturation of themes was achieved.

A majority of nominal groups (7/9) reported that gout was associated with worsening of other medical comorbidities, and hospitalizations. Gout flare was associated with worsening of blood pressure and heart conditions, and the use of gout medications was associated with lowered efficacy of medication for blood pressure control. Several patients were hospitalized with gout flares that also were associated with loss of optimal control of other comorbidities. A previous study reported that hospitalizations due to acute gout flares were longer in gout patients with renal failure, heart failure, osteoarthritis, or diabetes mellitus [34]. In addition, gaps in the inpatient management of gout [35] likely further contribute to a longer hospitalization. Thus, previous studies showed that there is a complex interaction of comorbidity and gout, which affect each other adversely and thus increase the risk of hospitalization and its duration. Nominal group participants noted that comorbidity management was affected adversely during an admission for gout flare, providing a further insight into mechanism of this problem/challenge.

Three patient nominal groups listed new disease diagnoses due to gout among their top concerns. Patients reported that since their gout was diagnosed, they had received new diagnoses of kidney disease, hypertension, heart conditions, sleep apnea and kidney stone (urate stones and calcium) disease, which they did not have before the diagnosis of gout. They believed that gout was the reason for them to be developing these new diseases. A patient said: “Gout could lead to heart disease, diabetes, cholesterol problems and atherosclerosis”. Hypertension, kidney disease, kidney stones, diabetes mellitus, heart disease and heart failure are common comorbidities in people with gout and hyperuricemia [8], as is metabolic syndrome [2]. Patients likened the urate crystals to “razor knives that cut through the tissues”, a mechanism they thought could lead to some of these conditions.

Three groups reported worsening of anxiety and depression due to gout. Patients reported both the negative effect of gout flares on anxiety and depression, their inability to concentrate during a gout flare, and the worry/anxiety associated with having a gout flare at night. This study provides insight into why depression may be more prevalent in patients with gout who have frequent gout attacks or attacks in multiple joints [36].

The focus of our study was to assess the effect of gout or its treatment on other comorbidities and their treatment. Several groups ranked the overall effect of gout on general wellbeing, daily activities, and gout medication-related important issues, rather than the impact on a specific disease. Since our broad research objective was to truly assess the impact of gout or its treatment, we did not force patients or groups to only choose a specific disease when they felt more appropriate choosing the effect on overall wellbeing, function, and daily activities. General well-being is an extremely important aspect of patient exprience, and in most instances represents an overall summative and cumulative effect of various diseases, their treatments and complications, experienced by the patient at the current time.

All nine groups reported side effects, real and perceived, from gout medications. Cumulatively, patients had experienced side effects from each gout medication. These included the following adverse events: NSAID-associated renal failure, GI bleeding, leg swelling and heart problems; colchicine-associated GI upset, dry mouth and renal function problems; corticosteroid-associated weight gain, GI upset, leg swelling, hip necrosis; allopurinol-associated nausea, allergic reaction, itching, rash, headache and brain fog; and febuxostat-associated heart problems were reported as symptoms/comorbidities resulting from gout treatments. Many patients also worried about the long-term side effects of gout medications even in the absence of any current side effects. A majority of nominal groups (6/9) reported the impact of gout on daily life and activities, the ability to work, and social activities. It is interesting that this emerged as a theme, despite the fact that the question was focused on other (health) conditions and their treatment. When discussed explicitly with the patients, they indicated that their overall ability to perform daily activities and function is more important than discussing another health condition; and that this ability reflected the impact of all their health conditions. Patients not only had difficult with daily activities and the tasks they enjoyed, but they also had to take sick days due to gout flares. Patients also had difficulty with social activities, such as going out to eat (due to dietary restrictions), watch a ballgame with family and friends, or attend important family or social gatherings (due to the uncertainty of a gout flare). The significant impact of gout on patients’ quality of life is well-known [12], and this study provides an in-depth insight into one mechanism for how and why this might actually happen.

These study findings must be interpreted considering the strengths and limitations of the study. Our findings may not be generalizable to all patients with gout, only those seeking health care. Over 80% of the patients were on urate-lowering therapy, a larger proportion than is reported in other gout cohorts, indicating that generalizability to populations with lower rates of treatment with ULTs may not be possible. The study sample more closely mirrors the epidemiology of gout in the USA [1] than many previous studies, which were focused on Caucasian men. Therefore, findings are generalizable to most patients with gout, who have a concomitant medical comorbidity. Another limitation is that patients described certain drug-disease interactions that have not been described previously such as allopurinol worsening diabetic control or gout medications (unspecified) rendering anti-hypertensive medication less effective, which are not consistent with previously known interactions/effects of these medications. These may represent the effect of other medications rather than the gout medications, i.e., patient misperception, or less likely, an unusual experience by a patient. We needed nine nominal groups to achieve theme saturation at least partially due to a small sample size for three nominal groups (n = 2–3 people in each group). Theme saturation can sometimes be achieved with a smaller number of groups. We conducted these small nominal groups realizing well that at least 4–5 people constitute a nominal group, since two of these three small nominal groups provided us with African-American patients, and the last nominal group helped us achieve saturation. The study strengths were the examination of a community clinic-based sample, inclusion of African-Americans and women with gout, and the focus on a single question.

## Conclusions

In conclusion, this study assessed the impact of gout on comorbidity. We found that gout and its treatment affect patient comorbidities and their management and daily living in several ways. Most of the highly ranked related themes led to body failure and/or negatively affecting patient’s social role by limiting identity-relevant performances with a progressive loss of self, thus fitting the proposed theoretical model. This study provides insight into the effect of gout on comorbidity. Studies need to explore the interrelationships of gout and comorbidity, and whether co-management can improve patient outcomes.

## Appendix

**Table 3:** All themes and related main discussions for the effect of gout or its treatment on comorbidity or its treatment from all nominal groups

Theme	Score
NGT1, Q2: 3 people: 3 male; 3 African-American (18 votes)	
A. When gout attacks it affects my movement, to walk and work	9
B. Before I got on the treatment, that I am on right now, the medicine made me sick (allopurinol associated nausea and allergic reaction)	5
C. Negative response to blood pressure treatment	2
D. Someone I know lost a lot of weight due to active gout that affected his other conditions	1
E. During a gout attack, gout test and potassium levels went high and had to be treated	1
NGT2 10 people; 4 male, 6 feamle; 4 white, 5 African-American, 1 Asian (60 votes)	
A. It affects your everyday body and brain functions, movement, physical or mental, makes me less independent	18
• Mobility –it got so bad, I had to wear brace • It affected my movement, can’t exercise, affects other conditions, I have • My knee gives away • Interfered with therapy, back pan and hip replacement	
K. Problem with misdiagnosis: It took a longer time frame for diagnosis of the actual problem in my joints, which is psoriatic arthritis, not gout	7
• General practitioner vs. Specialist: lack of complete understanding; general practitioner not concerned about sleep problems • Care is fragmented • I have psoriasis, now off medications, only on gout medications • Multiple diagnosis cause confusion	
F. More Depression/anxiety due to gout	4
E. Pain of gout increases your blood pressure and messes with the heart condition	4
• Swelling and inflammation affects blood pressure	
G. Specific diet advise differs between conditions, leading to contradictions, low protein for gout vs. high protein for other conditions	4
• They are tired of eating “my food”- I am the only one in the house who has to eat only a few type of foods • A lot of self-control • I loved sea-food; I haven’t had sea-food for a year, since I was diagnosed with gout I am down to chicken and broccoli • I love to eat seafood, now I can’t eat it all • Restriction of certain foods for gout makes it difficult • Have high potassium- by the time you merge the two lists of foods for both the conditions, you are hardly left with anything to eat- its’ chicken and broccoli – just chicken and broccoli all the time • With restriction for diabetes and gout, what is left to eat?	
B. I have kidney problems they had to take me off colchicine, since it was causing kidney problems	10
• They put me on Colcyrs which was interfering with my calcium level, they took me off colchicine • Affects my international issue (diverticulitis) • Medicine, Indocin, steroids and allopurinol, all caused hardening of the intestinal wall leading to colon resection • It causes brain fog- colchicine and allopurinol together, have trouble focusing, driving, walking • Sometimes colchicine and allopurinol gets me a little confused • Colchicine causes dry mouth, makes me thirsty • I was put on colchicine, caused gastric problems, has to be stopped	
G. I am diabetic, trying to avoid carbs; proteins that want to take to help regulate my diet, are problematic with gout	3
• Steroids increased my sugars and my diabetes • Prednisone complicated my diabetes • It affects my diet and eating regimen • I was prescribed fish oil for lowering cholesterol and can’t take that due to my gout	
J. Had problems with weight gain due to fluid retention due to gout	3
• Excessive weight gain due to steroids caused sleep apnea • Can’t exercise due to gout, that caused weight gain	
E. I have osteoarthritis, medicines can compliment each other	3
• I have pulmonary hypertension, heart failure, it does not help	
C. It interferes with other medications and makes me tired	2
• Medication interactions: • I take so much medicine	
H. Kidney stones were calcium oxalate only, now they are urate and calcium oxalate since I got gout	2
NGT3 Q2 (5 people); 2 male, 3 female; 4 white, 1 African-American (30 votes)	
I. My joints are so sore now all the time, wondering if they are damaged due to (gout) flares	8
• Sometimes unable to do activities of daily living • Sometimes can’t get to the doctor’s appointment • Can’t make it to the bathroom • I was off duty for 2 months due to gout flare	
B. Some of the gout medications such as prednisone can cause weight gain, diabetes and affect eyesight	8
• Bowel problems due to prednisone- pain in the belly • As far as the treatment goes, prednisone causes me to gain weight • I have been diagnosed with edema in the legs; I think medications (pain killers, prednisone) have something to do with it -could be the medication, doctor said • I had to take a lower dose of gout medication due to kidney problems • I am allergic to Neurontin, it caused face swelling and rash, when I was getting it for gout • NSAIDs cause bleeding • I was taking over the counter pain meds for gout, had kidney problems as a result	
E. I did not have high blood pressure until I was diagnosed with gout- not sure it caused it	9
• When you are having a flare, your BP goes way up • Heart rate was going up during the flare • I have heart conditions, I have several allergies and I have to take low doses of medications, like ibuprofen that mess them up	
J. Having gout makes me immobile, which makes it hard for me to loose weight	4
• Hard to exercise when foot hurts, have a flare • I was gaining weight at my first flare	
K. New diagnosis of rotator cuff for the last 3 weeks: Gout caused it to be misdiagnosed	1
A. Arthritis limits me in a lot of ways, could it be due to my gout?	0
• If I want to take a walk, I can’t go • Some days are better than others	
H. Kidney stones diagnosed after gout	0
• Had 2 huge ones removed- those were uric acid stones	
NGT4, Q2, 5 people; 4 male, 1 female; 1 white, 3 African-American, 1 Asian (30 votes)	
B. Gout treatment, in particular, prednisone caused weight gain	12
• Gout treatment caused new problems- acid reflux due to pain medications for gout • Other conditions limit what dose of gout medication I can take	
H. Gout putting you at risk for other conditions	7
J. Immobility and inability to exercise due to gout	5
K. Gout being confused with other conditions – I was tested for leg clots many times	4
E. Higher dose of allopurinol made my diabetes worse	2
NGT5; 4 people; 3 male, 1 female; 3 white, 1 African-American (24 votes)	
B. Gout medicines over long period time cause renal failure	19
• Indomethacin has contributed to my kidney disease- Indocin would reduce flares so I took it like candy • I would take it very day sometimes twice so that I could get to work in the military, started in 1983 and by 1993, they told me I had “shrunk kidneys”- I have been on dialysis since 2015 • Indocin caused 2 GI bleedings which cost me to need 4 units of blood • I tried taking Indocin but couldn’t take it, it made me crazy • Having gout flares all the time, took Indocin, and was diagnosed with stomach ulcers twice • They tell you to take it (Naprosyn) on a full stomach, I took it once on empty stomach and had severe vomiting • Prednisone caused renal failure “stage 4 kidney disease: my doctor put me on the prednisone, and they couldn’t get me off; every time they stop it, gout comes right back” • Prednisone caused me to develop necrosis of both hip joints • Woke up 1 day, I could not walk, they x-rayed me told me I had arthritis. Then I went to see my rheumatologist, he sent me to the other doctor, and he told me I had hip necrosis. They had to do a hip replacement, it got infected with Staph, then they had to do it again- I was in hospital for months with that, a lot of antibiotics • I couldn’t take Indocin due to stomach bleeds, so they put me on prednisone, and I had to have hip replacement for necrosis of the hip joint • Using medicines to subside gout has led to more arthritis which leads to taking more drugs and more side effects • If you have both gout and another type of arthritis, you have to take more drugs • I was put on Neurontin in addition to my gout medications	
H. Gout crystals caused me the “kidney problem”- kidney damage	3
• Crystals get filtered by your kidney • “They (crystals) are like razor knives, they cut your tissue – your kidney, other organs”	
L. The medication I take caused me to get my liver checked often	2
• I am on prednisone and also on cellcept for my scleroderma; on top of that Colcyrs makes my muscle enzyme go up sometimes- they are watching my liver tests and muscle enzymes	
NGT6 Q2, 7 people; 7 male; 4 white, 2 African-American, 1 Hispanic (42 votes)	
A. It affects my ability to work	12
• Totally immobile and can’t go to work with the flare • Happened after I retired, so no work issues, but bothered at home • “No body goes to work with gout, you have to be wheel-chaired in”	
L. Very expensive disease to control- Colchicine went from a few dollars a month to high price and co-pay overnight	8
• Colchicine is very expensive • The only prescription drugs I take are for prevention or active flares • The medicine for gout doesn’t cure you, you have to keep taking it • It’s a burden to take pills for gout • Medicines I take for gout are just treating the symptoms	
B. Side effects	9
• Indocin can mess up your liver • Colchicine and me don’t agree • I am worried about long-term side effects • Had kidney function deterioration due to Naprosyn, they put me on colchicine • I took big doses of Naprosyn for years, compromised my kidney function, numbers went south- it was 500 mg 2-3 times a day for 15 years • I worry about side effects and about taking these with other medications	
J. It affects my ability to do my work-out regimen, I work out 3-4 times a week	5
• During an attack, you don’t do anything • Feet are stiff during an attack • I work out to keep my weight in check, to feel better, for my neuropathy and to stay fit • When work out regimen changes, it also can bring out an attack	
F. It affects my ability to concentrate and stay focused	3
• When I am in such abstract pain, I can’t do anything	
L. Frequent regular visits to the doctor and the lab to monitor various levels of everything to adjust medications - Access to doctors is an issue, when I have to travel, if something happens, whom do I call?	3
• I also have common variable immunodeficiency and when I needed antibiotics, had to choose between antibiotic for infection versus colchicine for gout- I couldn’t take both at the same time, my doctor told me not to	
E. It totally incapacitates me and causes me to be hospitalized	1
• I was hospitalized because of my gout • I was hospitalized twice due to my gout (another patient) • I had to come off my large dose of Naprosyn for Pseudogout and gout, stopped due to surgery, then blew up my body at 3 places (knee, arm, foot) with gout flare, they had to call hospital consult and I stayed in the hospital 3 days longer than planned.	
I. Used to have Achilles tendinitis which was due to gout- with colchicine it’s gone	1
• Had one surgery for my broken ankle and then others due to arthritis I had due to my gout crystals damaging my joints • Had to have 2 surgeries due to gout • Broke both my ankles playing football- they keep seeing the break in my ankle on X-rays years after, wondering if that it due to the gout	
NGT7 Q2, 6 people; 1 male, 5 female; 3 white, 3 African-American (36 votes)	
A. Since you can not walk because of your pain, you are going to gain weight, and it will impact heart, lungs and body conditioning	19
• I need to exercise, my knees give out • I can not walk • Can’t play with grand kids • When you can’t walk, you are becoming the “Stale old thing” • “The more you sit, the more you want to sit” • My gout has caused me not do the things that I normally want to do like shopping and housekeeping • I love working in my yard, can’t do it now • It makes me feel old, didn’t have energy, physical flexibility • Can’t do what you want to do • You have a 30-year old son, doing everything that you can’t do anymore, he wants you to speed up or get out of his way- I used to be able to do all that when I was young and didn’t have gout, and now I can’t do that • You are afraid that you won’t be able to get back, if you walk too far because “your foot is killing you” and you wonder if someone can drive by and pick you up and drop at your home	
F. Makes me depressed	7
• Used to take vacation, and go over the U.S. with my husband all the time and we don’t do that anymore, we are afraid, might get sick	
E. It affects my eczema, when it is (gout flare) in my foot, the foot is itching all the time, and it bleeds if I itch it.	7
• Doctors haven’t told me that I have any other problem than gout • Take BP pills, but it does not come down- is that due to my gout? • When gout under control, BP is lower • Pain is better, BP is lower • Febuxostat treatment takes care of any arthritis related pain- no other treatment is required, no pain in the knee anymore	
B. A lot of gout medications affect kidneys and liver, especially the pain medication, so you can’t take a lot of pain medications	3
• I love Aleve, but I don’t take it every day • My liver function was up, when I took a lot of Aleve -I take Mobic low dose and I don’t have any side effect • Taking the medication (allopurinol) adds to your headache: contributes to the impact of all the medications • A lot of medications cause allergy- Allopurinol caused rash and itch • Allopurinol causes me stomach ache, headache, makes me feel bad	
G. Gout interfering with healthy eating; I can’t eat shrimp, cabbage, dry beans	0
• If you goggle, there a lot of foods that cause gout	
NGT8 Q2, 2 people; 1 male, 1 female; 2 African-American (12 votes)	
B. Gout medication – I was allergic to allopurinol	7
• I took allopurinol and broke out in a rash all over my body and inside of the body- I had to be in the hospital for a week, they had to treat me; now I take Uloric, and have no problem • Can not be out in sun, when you take colchicine- they told me not to do that, I haven’t had anything, but I don’t want to do it • Medication that I was taking for my gout (pain medication), treatment is too strong for my kidneys • I can’t take any arthritis pain medication due to my kidneys • They took me off naproxen, can’t take any pain medication other than Tylenol • Has a problem with my left kidney- the medication scarred my kidney, they said it was too much ibuprofen and Aleve	
C. Some of the medicines I can not take with my gout medicine- they interact	5
• Gleevac and colchicine interact, my doctors told me I can not take them together, so it’s a problem • Because of these gout medicines, I can not take medicines with grape fruit juice; I used to take all medicines with grape fruit juice, they told me not to take colchicine with grape fruit juice, so I have to take everything with orange juice now	
NGT9; 3 people; 3 male; 3 white (12 votes; 1 patient left before the vote due to transportation issues)	
H. Gout is causing other health conditions	4
• I think it (can) lead to heart disease, diabetes, cholesterol problems, artery stiffness and clogging • We don’t have as much activity as such, with gout you can even less • “A ship that sits at the harbor will sink”	
E. It has affected sleep and that affects my overall health	2
• Flares as well as when the gout hurts all the time outside of the flare	
B. At the back of my mind, I am concerned about the fact that the medicine is affecting my body	1
• All medicine is designed to be one thing, but you know it could cause long-term side effects • That bothers me as well; I was on Uloric and I was worried about heart problems- They thought I was having a heart attack and I stopped it after that, and am not taking any medicine, just cherry extract now	
A. Limits going to work, activity and going out	5
• When my gout flares, I can not go to work • It’s like age, “shuts you down” • Luckily, I am retired, but it interferes with going to other places • “Overall limits your experience of life” • It limits socializing • Used to enjoy going to Nashville, we cut down on the trips since my gout • Limits whether I can drive somewhere and go eat • Would always go with wife for shopping, now can’t do that • Not going to the movies anymore, don’t know when it will start • “It’s like walking on glass to get to your car- would you do that, or would you rest at your home?” • Affects vacation - I always get insurance on my trips, don’t know what will happen • If something happens (during vacation), no way to recover • I used to snow ski; now I just don’t go • On the social side, it has affected the activity of having a meal with friends and family • My son and daughter-in-law like to eat food high in fat; they cut back because of me, and now we have to pick a place that we can all eat at- it affects social life • It affects social life of my family, since they have to cut down on going out, and eating out • Before gout, I walked 4.5 miles a day, can it do that any more, restricted my activity • You just can not carry out activities, need for your shoulder, and durability • It was a part of my job, I walked a lot • I was able to maintain my cholesterol with my activity • It affects my daily activities around the house • Can’t work in the yard, can’t clean the gutters- I used to enjoy doing that • I am borderline diabetic, it limits exercise I can do	
I. Joints wear out quicker because of gout	0
• I had two joints repaired, in wrist and ankle • I went bowling and then woke up next day, it wasn’t so good (in my joints) • Things like throwing football, Now I throw underarm, can not do overarm anymore, because of my shoulder • You can shoot the basketball, but you can’t get the jump • Used to water ski- no more since I was diagnosed with gout • Lack of joint mobility	
F. Stress/anxiety due to gout	0
• A joint attack I could think of, experienced psychological side effects • During the day, no problem, but I wake up so many times during the night, just afraid it’s going to come back	
L. It has affected “my time”- You have to spend a lot of time with doctors and getting lab tests done	0
• It affected my time; I am seeing my doctors for gout every month	

Each patient gave 3 points to the highest rank, 2 points to the second highest and 1 point to the third highest rank. In most cases, total votes is the number of patients × 6, except in one case when the patient left before voting, due to transportation issues

## Abbreviations

BBC: Body, biographical time, and conceptions
ICD-9-CM: *International Classification of Diseases, ninth revision, common modification*
GI: Gastrointestinal
NGT: Nominal group technique
NSAIDs: Non-steroidal anti-inflammatory drugs
QoL: quality of life
UAB: University of Alabama at Birmingham
ULT: urate-lowering therapy
